# WNT pathway in focal cortical dysplasia compared to perilesional nonlesional tissue in refractory epilepsies

**DOI:** 10.1186/s12883-023-03394-1

**Published:** 2023-09-26

**Authors:** Daniel R. Marinowic, Gabriele G. Zanirati, Fernando A. C. Xavier, Fábio Jean Varella, Sofia Prates da Cunha Azevedo, Isadora Machado Ghilardi, Normando G. Pereira-Neto, Marco Antônio Eduardo Koff, Eliseu Paglioli, André Palmini, José Garcia Abreu, Denise C. Machado, Jaderson C. da Costa

**Affiliations:** 1 Brain Institute of Rio Grande do Sul (BraIns), Pontifical Catholic University of Rio Grande do Sul, Porto Alegre, Brazil; 2 Graduate Program in Medicine and Health Sciences, Medical School, Pontifical Catholic University of Rio Grande do Sul, Porto Alegre, Brazil; 3 Graduate Program in Medicine, Pediatrics and Child Health, Medical School, Pontifical Catholic University of Rio Grande do Sul, Porto Alegre, Brazil; 4 Graduate Program in Biomedical Gerontology, Medical School, Pontifical Catholic University of Rio Grande do Sul, Porto Alegre, Brazil; 5 Epilepsy Surgery Program, São Lucas Hospital, Pontifical Catholic University of Rio Grande do Sul, Porto Alegre, Brazil; 6 Biomedical Science Institute - Universidade Federal do Rio de Janeiro, Rio de Janeiro, Brazil

**Keywords:** Focal cortical dysplasia, Wnt pathway, Epilepsy, Perilesional tissue, NAAC

## Abstract

**Background:**

Focal cortical dysplasia (FCD) is a malformation of cortical development that causes medical refractory seizures, and one of the main treatments may be surgical resection of the affected area of the brain. People affected by FCD may present with seizures of variable severity since childhood. Despite many medical treatments available, only surgery can offer cure. The pathophysiology of the disease is not yet understood; however, it is known that several gene alterations may play a role. The WNT/β-catenin pathway is closely related to the control and balance of cell proliferation and differentiation in the central nervous system. The aim of this study was to explore genes related to the WNT/β-catenin pathway in lesional and perilesional brain tissue in patients with FCD type II.

**Methods:**

Dysplastic and perilesional tissue from the primary dysplastic lesion of patients with FCD type IIa were obtained from two patients who underwent surgical treatment. The analysis of the relative expression of genes was performed by a qRT-PCR array (super array) containing 84 genes related to the WNT pathway.

**Results:**

Our results suggest the existence of molecular alteration in some genes of the WNT pathway in tissue with dysplastic lesions and of perilesional tissue. We call this tissue of normal-appearing adjacent cortex (NAAC). Of all genes analyzed, a large number of genes show similar behavior between injured, perilesional and control tissues. However, some genes have similar characteristics between the perilesional and lesional tissue and are different from the control brain tissue, presenting the perilesional tissue as a molecularly altered material.

**Conclusion:**

Our results suggest that the perilesional area after surgical resection of tissue with cortical dysplasia presents molecular changes that may play a role in the recurrence of seizures in these patients. The perilesional tissue should receive expanded attention beyond the somatic mutations described and associated with FCD, such as mTOR, for example, to new signaling pathways that may play a crucial role in seizure recurrence.

**Supplementary Information:**

The online version contains supplementary material available at 10.1186/s12883-023-03394-1.

## Background

Focal cortical dysplasia (FCD) is a malformation of cortical development (MCD) characterized by intracortical architectural abnormalities associated or not with cytomegalic neurons and balloon cells [1, 2]. It is a very common cause of refractory epilepsy both in children and adults, hence being highly represented in epilepsy surgery series [3].

Although the histopathological common denominator of FCD is ‘dyslamination’, a term broadly referring to variable disarrangements of the normal cortical architectural pattern, its etiology is likely related to the presence and type of associated cellular abnormalities, as described in several classifications [2, 4, 5]. Particularly, type II FCD – characterized by the additional presence of dysplastic neurons associated (FCD IIB) or not (FCD IIA) with balloon cells - has been shown to be associated with somatic and germline mutations in genes related to AKT, mTOR and PI3K cell signaling pathways controlling cell growth and intracortical positioning [6]. Such discoveries led to further probing of abnormalities in other signaling pathways in FCD, such as the WNT/β-catenin signaling pathway, which is also crucial for controlling embryonic development, through the regulation of cellular differentiation, migration, proliferation and apoptosis. Since β-catenin is a protein responsible for cell‒cell adhesion and a component of the cadherin protein complex [7, 8], the WNT pathway could potentially also be involved in the etiology of FCD.

Interestingly, an often-neglected aspect when studying the molecular genetics of FCD is that beyond the lesion visible on magnetic resonance imaging (MRI), normal-appearing adjacent cortex (NAAC) may also have a lower epileptogenic threshold. Such abnormality is often indexed by epileptogenic discharges recorded over NAAC during acute electrocorticography (ECoG) and may or may not be associated with some degree of dysplastic abnormalities on histopathology. Thus, it is of clinical interest to study the WNT/β-catenin signaling pathway in NAAC of FCD as it may guide post-operative treatment with antiseizure medications and anticipate prognosis regarding seizure control.

Here, we study the WNT pathway in two patients operated for FCD IIA and compare abnormalities in the MRI-visible dysplastic tissue with both NAAC and normal cortex tissue obtained of patients subjected to non-selective amygdalohippocampectomy.

## Methods

### Ethics statement

This study was approved by the Committee of Research Ethics of the Pontifical Catholic University of Rio Grande do Sul (CAAE: 19776619.9.0000.5336 approval number: 3.577.035). Written consent was obtained from the parents of Patients 1 and 2 (who are underage) before they were enrolled in the study, according to Resolution n° 466/12 of Brazilian National Health Council.

### Characterization of patients

**Fig. 1:**
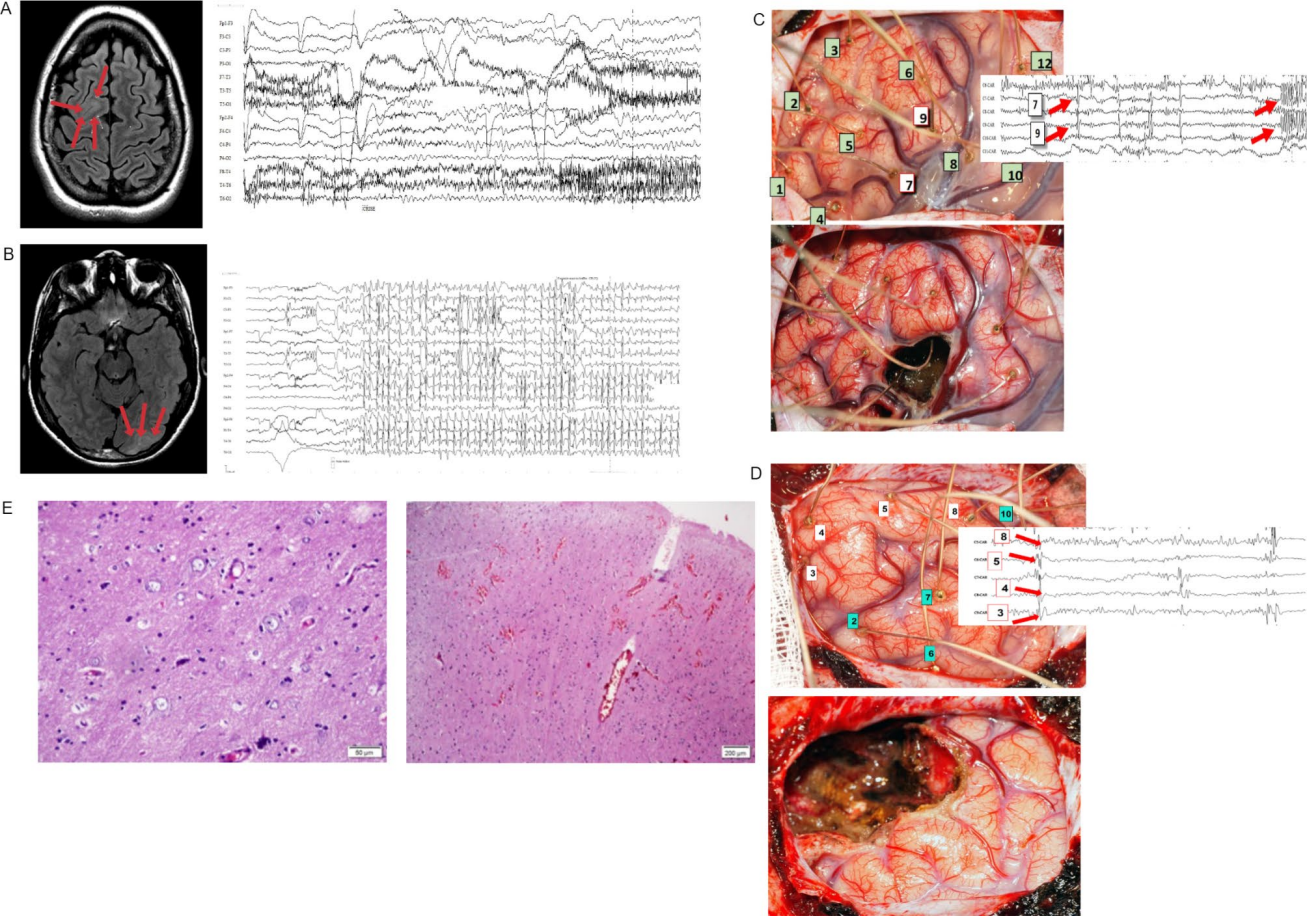
Clinical, intraoperative and anatomopathological features. Preoperative magnetic resonance imaging (MRI) and electroencephalogram of patient 1 **(A)** and patient 2 **(B)**. Arrows on MRI indicate the cortical area with dysplastic. In patient 1 **(A)**, MRI showed blurring and signal alteration of the cortico-subcortical interface of the posterior segment of the right superior frontal gyrus. MRI of patient 2 **(B)** depicted an area of cortical thickening signal alteration and blurring of the cortical-subcortical interface in the left occipital lobe. **C)** Cerebral cortex during intraoperative electrocorticography with the indication of each electrode placed on the surface of the cortex before and after surgical resection for patient 1 and 2 respectively **(D)**. Arrows focus on areas of epileptogenic activity. **C)** Histological section stained with hematoxylin and eosin of the lesion area showing the lack of the cortical lamination, dysmorphic and giants neurons, without presence of balloon cells, characterizing a Cortical Dysplasia Type IIa.

The tissue of two patients with FCD IIA and three controls histologically proven normal temporal neocortex was obtained at surgery for refractory epilepsies (Fig. 1). The control cortical tissue was obtained from patients with Temporal Lobe Epilepsy associated with Hippocampal Sclerosis (TLE-HS) submitted to anterior temporal lobectomy. Brief clinical characterization is followed by description of the molecular analyses.

#### Patient 1

This 17-year-old patient started with seizures at age 10 characterized by head turning to the right side and extension of the right and flexion of the left arm. Seizures were uncontrolled by anti-seizure medication and MRI showed blurring and signal alteration of the cortico-subcortical interface of the posterior segment of the right superior frontal gyrus compatible with FCD. Intra-operative acute electrocorticography (ECoG) showed very intense epileptiform discharges and recurrent electrographic seizures over the lesion and a right superior frontal gyrus resection was performed ‘en bloc’ allowing visual distinction between the dysplastic and non-dysplastic cortex. Histopathology confirmed FCD IIA. After 4years of follow up the patient remains seizure free.

#### Patient 2

This patient started with prolonged, frequent seizures at age 5, with head and eyes turning to the right side, groaning and sudden fall. Interictal EEG epileptic activity was very intense in the right temporo-occipital region. MRI depicted an area of cortical thickening signal alteration and blurring of the cortical-subcortical interface in the left occipital lobe, compatible with FCD. histopathology confirmed FCD IIA. After 4 years of follow up the patient is completely seizure free.

#### Sample collection

The routine procedures in the epilepsy surgery program for FCDs were followed. Craniotomy is performed to expose the clinically suspicious cortex based on previous imaging (MRI or PET-CT), video-EEG and seizure semiology. After craniotomy and dura-mater opening, acute electrocorticography (ECoG) is obtained and specific patterns indicative of intrinsic epileptogenicity points towards the FCD. The resection is performed with a minimal margin of what is thought to be normal adjacent tissue. Sequential acute ECoG is obtained, and resection continues if epileptogenic activity is observed in the non-eloquent cortex exposed. Samples of different areas are separately sent for histopathology analysis. NAAC sample was obtained following routine resection and no additional cortex was removed or manipulated for its obtention. All samples were immediately placed in RNA Later solution (Thermo Fisher Scientific, Massachusetts, EUA) and stored at -80 °C until RNA extraction. The clinical, intraoperative and anatomopathological features are showed in Fig. 1.

#### RNA extraction and generation of cDNA library

Total RNA was extracted from surgically resected brain tissues using the SV-Total RNA Isolation System (Promega, Madison, Wisconsin, EUA) following the manufacturer’s protocol. The extracted RNA was converted to cDNA using VILO MasterMix (Thermo Fisher Scientific, Massachusetts, EUA). cDNA was quantified in a NanoDrop to calculate the concentration needed for the qRT-PCR analysis.

#### Super array technique

The cDNA generated from the samples was added to a TaqMan® Array 96-Well Plate for the Human WNT Pathway. This super array contains 92 complementary primers from regions of genes related to the WNT pathway and 4 endogenous gene controls. The samples were amplified from the initial amount of 20 ng of cDNA for each sample. Real-time PCR was performed using StepOne Plus (Thermo Fisher Scientific, Massachusetts, EUA) equipment with the PowerUp Master Mix kit (Thermo Fisher Scientific). Initial results were analyzed in the Thermo Fischer Connect Platform for the generation of heatmaps.

#### Gene behavior analysis

Initially, heatmaps were generated using the calculated values of ΔCT (target gene-GAPDH) comparing tissue with dysplasia, perilesional tissue and control tissue. After this initial analysis, the ΔCT values were converted to a percentage scale to compose a visual presentation of the target genes in relation to the endogenous gene. After this conversion, it was possible to stratify the analyzed genes according to their behavior in relation to the 3 tissues. The subgroups created were: (i) genes with similar expression between the three tissues, (ii) genes with similar expression between the control and perilesional non-dysplastic tissue, (iii) genes with similar expression between the perilesional tissue and the dysplastic tissue greater than the control, (iv) genes with similar expression between the tissue perilesional tissue and lesion (dysplastic) tissue smaller than the control, (v) genes with progressively increasing expression in the perilesional tissue and dysplastic, and (vi) genes with similar expression between control and lesion and different from the perilesional tissue. Finally, we analyzed the fold change results comparing the tissues NAAC and Lesion using healthy cortical tissue as a control group and the values of fold change were grouped. The analysis of the results was performed using GraphPad Prism.

## Results

### Heatmap analysis

**Fig. 2:**
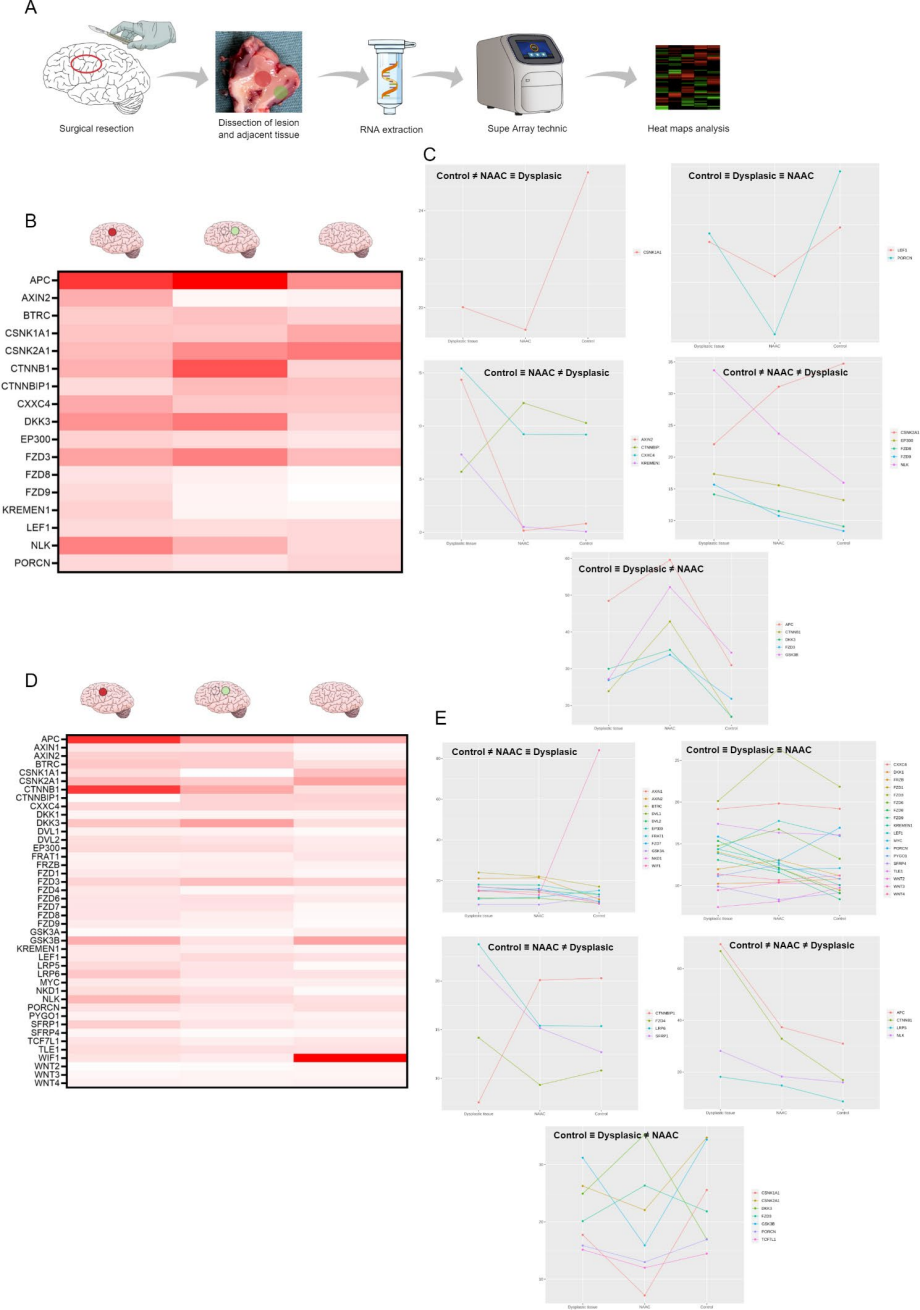
Analysis of the molecular profile of the WNT pathway in cortical tissue. **A)** Ilustration of methodology used to obtain the results of analysis of the expression profile of the genes of the WNT pathway using the super array technique in dysplastic and NAAC tissue. **B)** Comparison of ΔCT values of the genes analyzed by heat map of cortical tissue with dysplastic lesion, NAAC and control cortical for patient 1 **(B)** and patient 2 **(D)**. These different tissues are showed in the columns and represented by labeled red (dysplastic tissue), green (NAAC) and without labeled (control) in the illustrative brain image. **C** and **E)** Analysis of percentage of ΔCT of patiens stratifying the genes analyzed by the difference in behavior in the comparison of the 3 different tissues analyzed, thus being able to identify which genes present similarity and/or difference between the tissue with dysplasia and the adjacent tissue compared to the control. The separation of this genes allows observe the different presentation relationship with the genes expression comparing the three tissues, being watched genes with ΔCT values similar in the dysplastic, NAAC and control, and genes with different expression sometimes with control, NAAC and dysplastic tissue for patient 1 **(C)** and patient 2 **E)**. (≠ different / ≡ similar)

The heatmap illustrates the expression patterns of WNT pathway genes from dysplastic tissue, NAAC and control brain (Fig. 2). Each horizontal row represents the same gene product, and each vertical row represents the same tissue. The fluorescence ranged from high (red - maximum) to low (yellow - minimum) is indicated by the colored bar and reflects the degree of value of delta CT on a logarithmic scale. These values are also presented in a stratified way in the subgroups for a better visualization of the behavior of the delta CT values in each of the 3 tissues, as well as the relative expression values generated from the expression calculation (2 ^–ΔΔCT^) using tissue without dysplastic lesions as a control.

### Molecular analysis

In both patients, many genes related to the WNT pathway had distinct behaviors in the tissue from FCD, NAAC and normal neocortex controls.

**Fig. 3:**
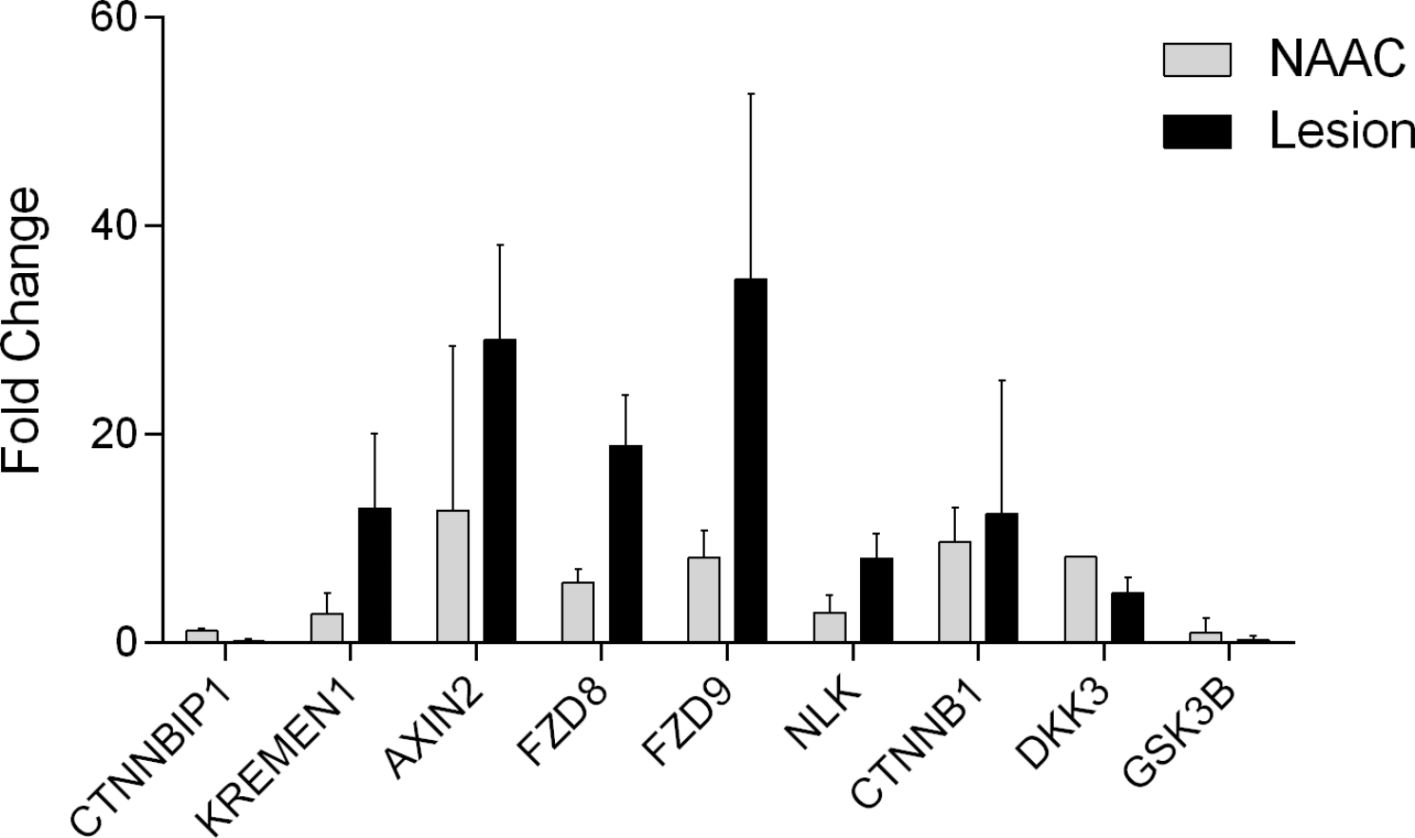
Analyze of the results by grouping the patients and comparing the values of Fold Change (FC). Relative quantification methodology was performed using 2^−ΔΔCT^ comparing dysplastic and NAAC brain tissues with control brain tissue. Some genes showed important difference in de fold change values in the NAAC and lesion tissue. Most of the genes analyzed showed a gradual and growing increase of fold change for NAAC and lesion in compare to control brain tissue

Analyzing the results by grouping the patients and comparing the values of Fold Change (FC), values were found with a gradual increase of FC, for example, for the KREMEN gene, the FC in NAAC was 2.76 times, and the lesion tissue was 12.93. The FZD8 gene showed FC of 5.83 times in NAAC and 18.9 in lesion. This difference in values was quite constant in the analyzed genes, highlighting the FZD9 gene with 8.16 times in NAAC and 34.9 times in the lesion tissue. No FC value showed a statistically significant difference between groups (Fig. 3).

### Patient 1

Some genes had similar log delta values in tissue with dysplasia, NAAC and healthy control tissue. However, the expression of other genes differed according to the tissue. For instance, the CTNNBIP1, CXXC4, KREMEN1 and AXIN2 genes showed very similar percentage values in the control tissue and in the NAAC but had significantly different in values in the FCD tissue. On the other hand, the percentage of delta value of the CSNK1A1 gene showed a similar value in the dysplastic and NAAC but was different in the control tissue. Interestingly, some genes showed graded contrasts, with significant percentage delta difference between control tissue and NAAC and even greater difference between NAAC and the FCD. Genes with this behavior included: EP300, FZD8, FZD9, NLK and CSNK2A1. APC, CTNNB1, DKK3, FZD3 and GSK3B genes showed changes in percentage delta values; however, there was a greater similarity between the FCD and the control tissue and a greater difference with the NAAC. (Fig. 2C).

**Fig. 4:**
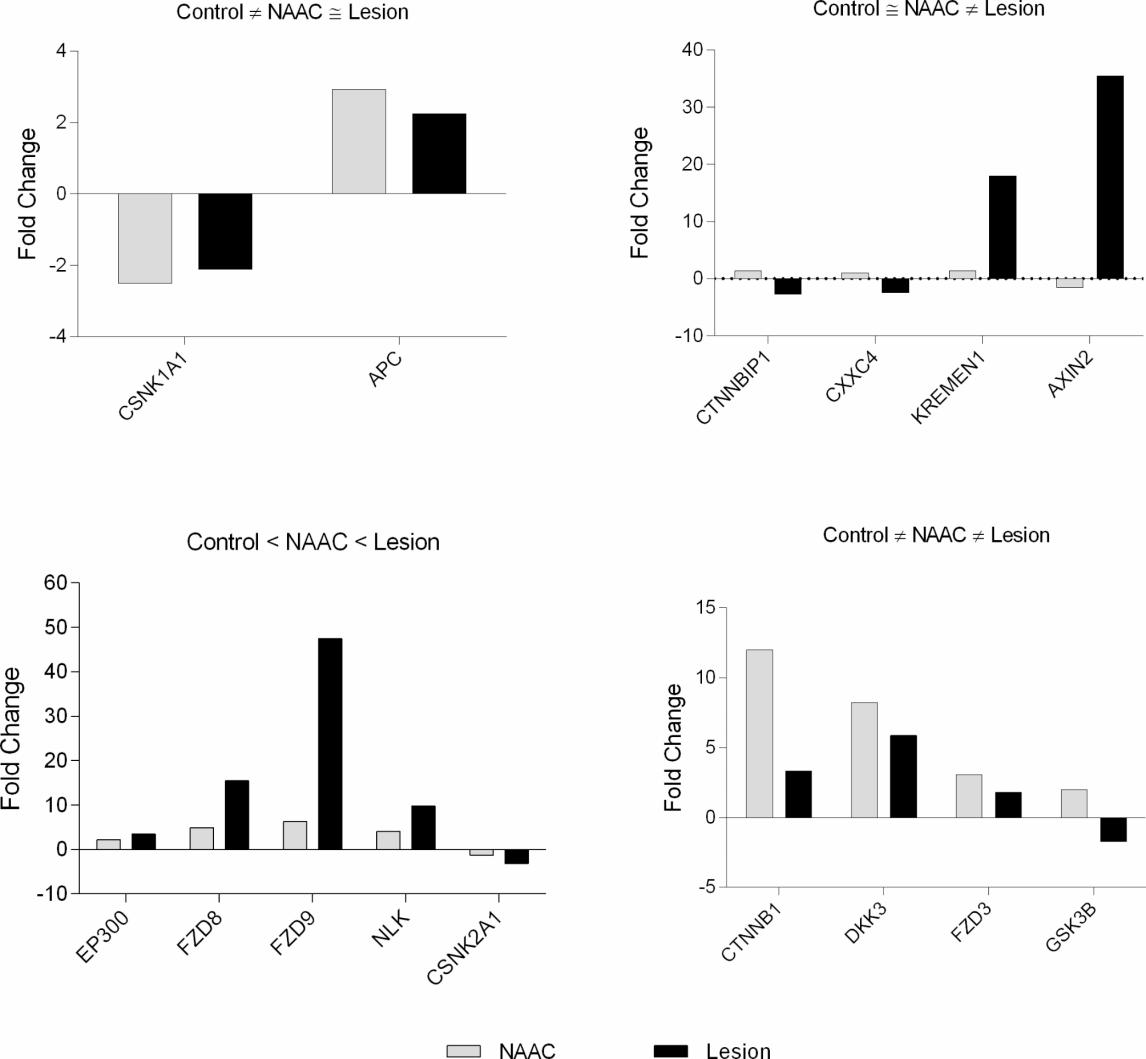
Analysis of gene expression of WNT pathway in patient 1. The analyzes were grouped according to the similarity or difference in gene expression. Relative quantification methodology was performed using 2-^ΔΔCT^ comparing dysplastic and adjacent brain tissues with control brain tissue

For a better understanding regarding the behavior of genes in the three different tissues, the analysis of relative expression (2^−ΔΔCT^) is presented using uninjured tissue as a control for relative expression (Fig. 4). A group of genes showed alterations in relative expression only in the dysplastic cortex. The CTNNBIP1 gene showed a relative expression 2.71 times lower than the control group, and the CXXC4 gene showed a relative expression 2.42 times lower. The KREMEN1 gene showed an 18-fold increase in relative expression in tissue with dysplasia when compared to the control. AXIN2 gene had a very expressive increase in the fold change value, reaching 35.5 times more gene expression compared to the control. Furthermore, the CSNK1A1 and APC genes showed an increase in relative expression both in the NAAC and in the dysplastic cortex, while for CSNK1A1 there was an increase of 2.49 times in the NAAC and 2.11 times in the FCD tissue and for the APC gene, 2.93 and 2.24 times increased, respectively.

Another group of genes showed a gradual change in relative expression in NAAC and control tissue. EP300 gene showed a 2.2-fold increase in the NAAC and a 3.48-fold increase in dysplastic tissue. FZD8 and FZD9 genes showed increases of 4.92 and 6.28 times, respectively, in NAAC and 15.45 and 47.47 times in tissue with dysplasia. In contrast, the NLK gene showed a similar behavior in the relative expression, being 4.11 times more expressed in NAAC and 9.78 times more expressed in tissue with FCD.

CTNNB1, DKK3 and FZD3 CTNNB1, DKK3 and FZD3 genes had higher expression in the NAAC when compared to dysplastic cortex.

### Patient 2

The heatmap analysis shows a wide variability in the expression of the analyzed genes. There were differences in the delta CT values of tissue with cortical dysplasia when compared to healthy brain tissue, but NAAC also showed differences in delta CT values when compared to healthy tissue and tissue with dysplasia. (Fig. 2D).

SFRP1, LRP6, FZD4 and CTNNBIP1 genes showed very similar percentage delta CT values in control tissue and NAAC and with a difference in these delta values in tissue with cortical dysplasia. Only the CTNNBIP1 gene showed a lower value of percentage of delta, while the other genes showed higher values. Moreover, some genes such as NKD1, FZD7, EP300, DVL2, DVL1, BTRC, AXIN2 and AXIN1showed similar percentage delta values in the dysplastic and NAAC, both with higher values than the control tissue. In a similar way, but with lower values of percentage of delta in relation to controls, WIF1, GSK3A, FRAT1 and CSNK2A1 genes had similar values in the FCD and control tissues. (Fig. 2E).

Similar to patient 1, a group of genes (NLK, LRP5, KREMEN1, FZD9, FZD8, FZD1, CTNNB1 and APC) showed a decreasing gradient in percentage of delta values from dysplastic tissue to NAAC to cortex from controls. Of interest, TCF7L1, DKK3, CSNK1A1, GSK3B, FZD3 e PORCN genes demonstrated a similar percentage of delta values in controls and dysplastic tissue, but both differed from NAAC (Fig. 2E).

Regarding the relative expression of control tissue, a group of genes showed alteration only in the dysplastic lesion: CTNNBIP1 showed a relative expression 326.74 times lower than the control tissue, FZD4 and LRP6 respectively had 4.58 and 4.93-fold increase, and SFRP1 showed a 9.48-fold increase in relative expression (Fig. 4). Additionally, some genes showed changes in relative expression both in tissue with dysplasia and NAAC. For instance, the AXIN1 gene showed a 12.38-fold increase in relative expression in NAAC and 12.62-fold in FCD tissue, whereas AXIN2 had a 23.88-fold increase expression in NAAC and 22.61-fold increase in dysplastic tissue. Similarly, DVL1 and NKD1 genes showed significant increase in relative expression values, from 5 to 50 times higher than control tissue.

**Fig. 5:**
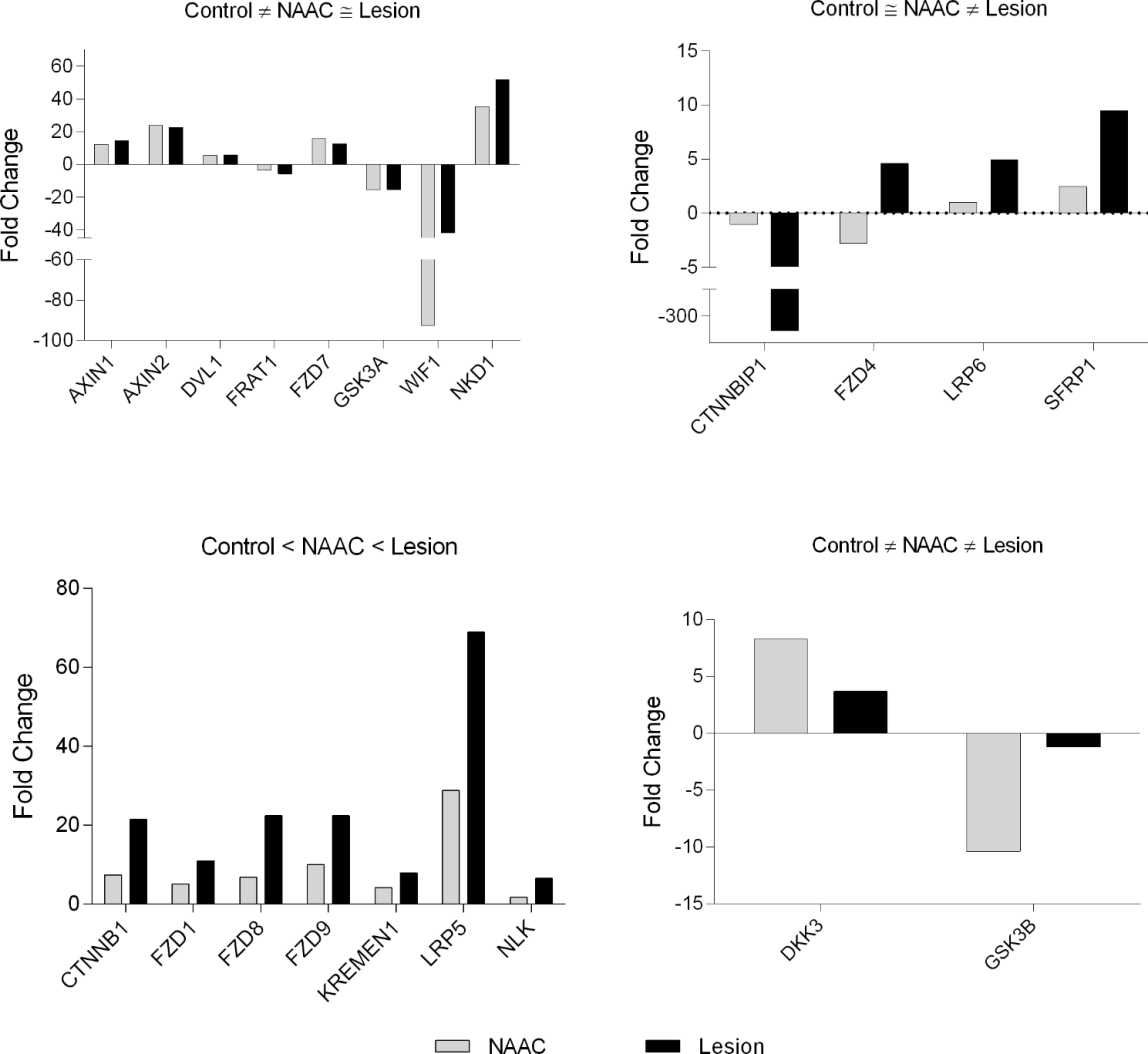
Analysis of gene expression of WNT pathway in patient 2. The analyzes were grouped according to the similarity or difference in gene expression. Relative quantification methodology was performed using 2-^ΔΔCT^ comparing dysplastic and adjacent brain tissues with control brain tissue

Importantly, some key genes such as GSK3A showed a lower relative expression with a 15.04-fold reduction in the NAAC tissue and 15.03 in the dysplastic lesion. WIF1 gene showed a very expressive reduction, being 92.4 times less expressed in NAAC and 41.96 times less expressed in tissue with dysplasia (Fig. 5).

Finally, relative expression analysis also allowed the identification of genes with gradual differences between dysplastic cortex, NAAC and control tissue, including CTNNB1, FZD1, FZD8, FZD9, KREMEN LRP5 genes. In contrast, Fig. 2D shows that DKK and GSK3B had an inverse relative expression, higher in NAAC than in the FCD. The DKK3 and GSK3B genes showed diffuse alterations between the NAAC and control tissues, and FCD tissue with reactive expression values less altered than the NAAC in relation to the control tissue (Fig. 4).

## Discussion

We have shown that the WNT molecular pathway has a complex behavior both in MRI-visible FCD IIA tissue and in the NAAC. Patterns of expression of many genes in FCD significantly differed both from NAAC and epileptic control tissue, whereas others were similarly expressed both in dysplastic and surrounding tissue but differed from the expression in control tissue. These results not only support the role of the WNT pathway in FCD, but open further avenues of research regarding molecular modifications in the cortex that surrounds a visible dysplastic lesion that is often responsible for seizure relapse following epilepsy surgery.

Success in epilepsy surgery for FCDs relies on correctly defining and fully resecting the epileptogenic zone (EZ), a challenging endeavor as shown by suboptimal surgical outcome in many patients [2, 9]. Several factors are implicated in suboptimal results, including the fact that FCD may have only subtle or no MRI abnormalities and that even in patients with clear imaging abnormalities, dysplastic tissue can either extend microscopically beyond the visible lesion, or alter the epileptogenic threshold of cortical adjacent tissue [10].

Several methods are available for the neurophysiological refinement of the EZ. In our patients, we used acute electrocorticography (ECoG) as we have shown that dysplastic cortex – and particularly FCD II – often displays intense, virtually continuous epileptiform discharges pointing to intrinsic epileptogenicity of the tissue [11, 12]. Following resective surgery, patients may remain seizure free in the long term, or seizures may recur. Early recurrence is most often due to incomplete resection of the EZ, and reoperation can be considered. However, the most intriguing scenario is delayed seizure recurrence following several years of seizure freedom, and this outcome remains blind spot in epileptology. Several factors may be associated with long-term outcomes in surgeries for FCDs. Completeness of resection, defined by MRI and scalp EEG, seems to be the most important independent variable associated with seizure freedom in both early and long-term follow up [13]. Given that, the first hypothesis for the late seizure relapse could be the incomplete resection of FCD’s whole extension, with remaining microscopic dysplastic abnormalities that eventually begin to produce independent seizures by a ‘running up’ phenomena. The second hypothesis is the emergence of a potential epileptogenic zone not clinically manifested before the first surgery. Third, the NAAC may not present dysplastic microscopic tissue, but could be related to a reduced epileptogenic threshold playing a role in a broader epileptic network – i.e., the NAAC is the potential epileptogenic zone itself.

This latter phenomenon may be related to a reduced epileptogenic threshold of NAAC, that then starts to generate seizures over time. However, what could reduce the epileptogenic threshold of the NAAC is unclear: in some patients there are remaining microscopic dysplastic abnormalities that eventually begin to produce independent seizures, but in other situations the tissue histopathology may not be dysplastic, and yet seizures may still recur.

We believe that molecular changes in both the obviously dysplastic and NAAC may concur to the epileptogenicity in patients with FCD IIA and relate to surgical prognosis in the short and long term. A similar classification in relation to perilesional tissue was presented in 2017 by Aran et al. based on an analysis of the transcriptome of tumor tissue and adjacent tissue from eight different types of tumors, showing for the first time the term NAT (Normal Adjacent Tissue). The researchers proved that the tissue adjacent to the tumor presents a unique intermediate state between tumor and healthy, and the analysis of gene expression and interaction between specific proteins revealed an alteration in signaling pathways shared between NAT and the tumor tissue, still listing 18 genes with specific activity in tissue adjacent to the tumor [14]. Recently, Guerrini et al. presented 2 patients with FCD type II with somatic mutation of the mTOR gene only in the dysplastic tissue. After repeated unilateral resections and eventual complete hemispherectomy, both began to manifest intractable seizures originating in the contralateral hemisphere. They suggested that the distribution of mutations along the mTOR pathway may relate to a potential risk of seizure recurrence, perhaps independently from the completeness of the resection of the lesion [15].

Approximately 60% of patients with FDC type II have some somatic mutation in genes related to the mTOR pathway, but the vast majority (80%) have rates of only 5% of mutated cells in the dysplastic tissue. The other patients with FDC type II do not have defined causes related to any mutation [16, 17]. Therefore, new signaling pathways should be explored to identify possible mechanisms of inhibition, control, or silencing, seeking a better understanding of the genesis of the epileptogenicity in FCD and surrounding cortex.

In their systematic review, Melo et al. (2021) analyzed a total of 648 patients with Focal Cortical Dysplasia, all with the characteristic of refractoriness to drug treatment and undergoing surgery for resection of the epileptogenic zone. For patients diagnosed with FCD type II, the most commonly affected gene was MTOR, which could represent up to 57.1% of the patients studied. All somatic MTOR variations were missense variants, leading to an amino acid and protein substitution. The article concludes that the pathogenic mechanism of FCD (genetic and epigenetic) and the discovery of new candidate genes are challenging areas that need a deep investigation [18].

A transcriptome study analyzed 68 mTOR pathway genes in brain tissue and blood in 17 patients with FCD type I and II. Comparison between patients with FDC type I and II showed differences in the expression of 12 genes. It is interesting to note that the principal component dimensionality reduction method demonstrated that samples of FCD type I and II could be aggregated into distinct clusters. In addition, the work points to the importance of genes not yet investigated, such as 3 negative regulations for cholesterol synthesis (HMGCS1, HMGCR and SQLE), described in this study [19]. Since FCDII pathogenesis relies on alterations of cell migration and differentiation as shown by the dysmorphic neurons, balloon cells and delamination, to explore the potential association of FCDII and Wnt/beta-catenin pathway, as it is known for regulating cellular differentiation [20–22] and cell migration [23, 24]. In fact, mTOR signaling also is thought to regulate both differentiation and migration. Hence, it is within reason to assume a correlation between both pathways [25–27].

We observed a similarity in gene expression (2^−ΔΔCT^) in the dysplastic tissue of both patients. In contrast, gene expression in adjacent peri-dysplastic tissue (NAAC) was distinct in each patient, although both differed from that of control tissue. Our results suggest the existence of a molecular alteration of some genes of the WNT pathway in tissue with dysplastic lesions and in peri-dysplastic adjacent tissue. The similar expression behavior of the AXIN1, AXIN2 and NKD1 genes suggests that the WNT pathway has a molecular profile of inactivation. A discrepant finding that can be explained by a compensatory mechanism of inactivation of the pathway is the robust negative regulation of WIF1 in the lesion and perilesional tissue, which is considered a potent negative regulatory gene and universal as a target of inhibition of the WNT pathway [28].

These findings suggest that FCD type IIA and NAAC have abnormal, often graded gene expression along the WNT pathway compared to control cortex. Interestingly, however, abnormalities in NAAC seemed less homogeneous, and it is important to note that specimens were obtained from different brain regions (frontal lobe in patient 1 and occipital lobe in patient 2). Differences in gene expressions between NAACs could be attributed to regional gene expression across different brain regions. Some studies have already presented a detailed profile of gene expression in the entire human brain, revealing spatial structures in hierarchical groups and associating robust modules of highly correlated genes with regions and functional associations [29]. Regarding the cortex, differences in cellular and molecular activity patterns are not well understood. Shin et al., 2018, evaluated different molecular markers of neural cells speculating their interregional variations in the human cerebral cortex, as well as the association of these interregions with cortical thickness. This study demonstrated that the interregional cortical thickness profiles are related to differences in gene expression, mainly associated with the three major cell types that explain 70% of the regional variation in cortical thickness [30].

This finding deserves further study as such NAAC seems crucial to understand surgical failures, particularly delayed post-operative seizure relapse. Further issues that will need to be explored in larger series include the relevance of epilepsy duration, age at surgery and frequency of epileptiform discharges, for gene expression.

In addition, because a functional neural circuitry requires homeostatic balance between proliferation of neural progenitors and triggers of cell differentiation, both largely dependent on physiological activation [31, 32], genetic alterations in peri-dysplastic tissue in patients with FCD may be associated with seizure relapses. More specifically, alterations in the control of cell proliferation and differentiation mediated by imbalances within the WNT pathway may lead to seizure relapse after resection, due to failure of mechanisms of neurogenesis stimulated by the resection itself.

Our results additionally suggest that it will be interesting to look for the role of functional neuroimaging studies (i.e., FDG-PET) in the identification of abnormal peril-dysplastic tissue that could later be correlated with abnormalities in gene expression [33]. Moreover, animal models show changes in the epileptogenic potential of brain tissue adjacent to the surgically removed dysplasia. Dysplasia itself can reorganize brain circuits and resections may lead to electrical imbalances in epileptic networks [34] As alluded to above, epileptic discharges may also lead to structural and functional cortical changes, increasing the tendency to epileptogenicity in secondary areas not primarily resected [35].

In recent years, the WNT pathway has gained considerable attention in seizure-induced neurogenesis and its consequent neuronal homeostasis after a seizure. On one hand, initial seizures increase neurogenesis in the acute phase of epilepsy; on the other hand, in the chronic phase of the disease, there is a reduction in neuronal proliferation, which is an extremely important issue for the optimization of therapies using the WNT pathway as a target of activation or inhibition [36].

## Conclusions

Our results suggest that the perilesional area after surgical resection of tissue with cortical dysplasia presents molecular changes that may play a role in the recurrence of seizures in these patients.These changes can be triggered by the injury caused during the resection and the consequent attempt to recover this tissue, potentiating its malfunction and being able to corroborate to assume an epileptogenic role a few months after the surgery procedure. Two important strategies for optimizing the surgical procedure could be elaborated from this hypothesis: (a) monitoring at the molecular level to delineate the surgical resection margin and, therefore, resection with greater extension and safety in terms of recurrence; and (b) therapy targeting the WNT pathway in the perilesional region during the surgical course. The development of WNT-inhibiting therapies has been a topic of great interest in the field of clinical oncology and medical chemistry [36–39]. The aberrant activation of WNT signaling cascades is involved in cancer pathogenesis, therapies that inhibit or potentiate WNT signaling are necessary for future implementation of precision medicine with targeted therapies in human diseases.

Several limitations of this study should be noted. First, the small number of patients prevent deeper statistical analysis and external validation. Second, these results rely on gene expression through the super array technique, lacking confirmation by qRT-PCR, immunohistochemistry, structural and inhibitory markers, among others and FCDs itself also represent a heterogeneous disease underlying a complex molecular condition.

This notwithstanding, our results suggest that FCD does not present a “well defined border” in terms of gene expression and signaling pathways. Perilesional tissue displays a distinct signaling pathway than the FCD and should receive expanded attention beyond the somatic mutations in the mTOR pathway. This can play a crucial role as a potential epileptogenic tissue and aid the understanding of seizure recurrence in FCDs.

### Electronic supplementary material

Below is the link to the electronic supplementary material.

Supplementary Material 1

Supplementary Material 2

## Data Availability

The datasets used and/or analysed during the current study are available from the corresponding author on reasonable request.
