# Supplementary material for: WNT pathway in focal cortical dysplasia compared to perilesional nonlesional tissue in refractory epilepsies
Source: BMC Neurol. 2023 Sep 26;23:338. doi: 10.1186/s12883-023-03394-1 (PMC10521408; doi:10.1186/s12883-023-03394-1)
Supplement: Supplementary file 1 — Supplementary Material 1 [file 12883_2023_3394_MOESM1_ESM.docx]

Patient 1 adjusted delta values

Control ≠ NAAC = Dysplasic

|  | Lesion | NAAC | Control |
| --- | --- | --- | --- |
| CSNK1A1 | 20,02002 | 19,09126 | 25,57545 |

Control = NAAC = Dysplasic

|  | Lesion | NAAC | Control |
| --- | --- | --- | --- |
| LEF1 | 15,69859 | 15,10574 | 15,94896 |
| PORCN | 15,84535 | 14,10039 | 16,92047 |

Control = NAAC ≠ Dysplasic

|  | Lesion | NAAC | Control |
| --- | --- | --- | --- |
| CTNNBIP1 | 15,6912 | 22,16312 | 20,28398 |
| CXXC4 | 25,41296 | 19,22707 | 19,19386 |
| KREMEN1 | 17,31302 | 10,49759 | 10,06036 |
| AXIN2 | 24,3546 | 10,14816 | 10,81081 |

Control ≠ NAAC ≠ Dysplasic

|  | Lesion | NAAC | Control |
| --- | --- | --- | --- |
| EP300 | 17,34605 | 15,55452 | 13,22751 |
| FZD8 | 14,13428 | 11,49293 | 9,090909 |
| FZD9 | 15,67152 | 10,75384 | 8,368201 |
| NLK | 33,67003 | 23,69668 | 15,97444 |
| CSNK2A1 | 22,03128 | 31,08486 | 34,72222 |

Control = Dysplasic ≠ NAAC

|  | Lesion | NAAC | Control |
| --- | --- | --- | --- |
| APC | 48,44961 | 59,59476 | 30,95975 |
| CTNNB1 | 23,89486 | 42,8449 | 16,89189 |
| DKK3 | 29,98501 | 35,12469 | 16,97793 |
| FZD3 | 26,89618 | 33,78378 | 21,83406 |
| GSK3B | 27,1813 | 52,19207 | 34,36426 |
