# Supplementary material for: WNT pathway in focal cortical dysplasia compared to perilesional nonlesional tissue in refractory epilepsies
Source: BMC Neurol. 2023 Sep 26;23:338. doi: 10.1186/s12883-023-03394-1 (PMC10521408; doi:10.1186/s12883-023-03394-1)
Supplement: Supplementary file 2 — Supplementary Material 2 [file 12883_2023_3394_MOESM2_ESM.docx]

Patient 2 adjusted delta values

Control ≠ NAAC = Dysplasic

|  | Lesion | NAAC | Control |
| --- | --- | --- | --- |
| AXIN1 | 14,8368 | 14,32665 | 9,425071 |
| AXIN2 | 21,0482 | 21,40411 | 10,81081 |
| BTRC | 23,97507 | 22,04586 | 17,03578 |
| DVL1 | 11,42335 | 11,34173 | 8,865248 |
| DVL2 | 16,90617 | 15,06705 | 13,17523 |
| EP300 | 18,0538 | 17,80944 | 13,22751 |
| FRAT1 | 11,01079 | 11,83572 | 15,19757 |
| FZD7 | 15,11944 | 15,99 | 9,737098 |
| GSK3A | 8,206138 | 8,200082 | 12,12121 |
| WIF1 | 15,19526 | 12,95169 | 84,03361 |
| NKD1 | 16,88619 | 15,43448 | 8,605852 |

Control = NAAC = Dysplasic

|  | Lesion | NAAC | Control |
| --- | --- | --- | --- |
| CXXC4 | 19,15342 | 19,82947 | 19,19386 |
| DKK1 | 10,22495 | 10,40691 | 10,81081 |
| FRZB | 11,95457 | 13,07702 | 11,19821 |
| FZD3 | 20,12072 | 26,35046 | 21,83406 |
| LEF1 | 14,34514 | 17,73679 | 15,94896 |
| MYC | 13,86194 | 11,89768 | 12,0919 |
| PORCN | 15,86546 | 12,97859 | 16,92047 |
| PYGO1 | 11,13834 | 12,46727 | 10,81081 |
| TLE1 | 17,38526 | 16,31854 | 16,05136 |
| WNT2 | 7,423354 | 8,104384 | 9,746589 |
| WNT3 | 9,445546 | 10,34447 | 10,07049 |
| WNT4 | 11,37656 | 10,66553 | 11,21076 |
| SFRP4 | 9,891197 | 8,331251 | 9,13242 |
| FZD6 | 14,76451 | 16,7392 | 13,21004 |
| FZD1 | 14,00756 | 12,11387 | 9,442871 |
| FZD8 | 15,34213 | 12,12415 | 9,090909 |
| FZD9 | 13,0736 | 11,59958 | 8,368201 |
| KREMEN1 | 14,36369 | 12,70164 | 10,06036 |

Control = NAAC ≠ Dysplasic

|  | Lesion | NAAC | Control |
| --- | --- | --- | --- |
| CTNNBIP1 | 7,528987 | 20,09243 | 20,28398 |
| FZD4 | 14,17635 | 9,327488 | 10,81081 |
| LRP6 | 23,76426 | 15,39883 | 15,36098 |
| SFRP1 | 21,57963 | 15,15841 | 12,69036 |

Control ≠ NAAC ≠ Dysplasic

|  | Lesion | NAAC | Control |
| --- | --- | --- | --- |
| APC | 69,44444 | 37,31343 | 30,95975 |
| CTNNB1 | 66,75567 | 32,87311 | 16,89189 |
| LRP5 | 18,13894 | 14,76233 | 8,605852 |
| NLK | 28,12148 | 18,21825 | 15,97444 |

Control = Dysplasic ≠ NAAC

|  | Lesion | NAAC | Control |
| --- | --- | --- | --- |
| CSNK1A1 | 17,7305 | 7,163324 | 25,57545 |
| DKK3 | 24,91901 | 35,27337 | 16,97793 |
| TCF7L1 | 15,14005 | 11,99185 | 14,45087 |
| CSNK2A1 | 26,2674 | 22,06045 | 34,72222 |
| GSK3B | 31,21099 | 15,89825 | 34,36426 |
| FZD3 | 20,12072 | 26,35046 | 21,83406 |
| PORCN | 15,86546 | 12,97859 | 16,92047 |
